# Concurrent germline and somatic pathogenic BAP1 variants in a patient with metastatic bladder cancer

**DOI:** 10.1038/s41525-020-0121-8

**Published:** 2020-03-23

**Authors:** Megan E. Tesch, Justin A. Pater, Gillian Vandekerkhove, Gang Wang, Kristin Binnington, Alan I. So, Alexander W. Wyatt, Bernhard J. Eigl

**Affiliations:** 1 Department of Medical Oncology, British Columbia Cancer Agency, Vancouver, BC Canada; 2 Department of Pediatric Oncology, Dana-Farber Cancer Institute, Harvard Medical School, Boston, MA USA; 3 Vancouver Prostate Centre, Department of Urologic Sciences, University of British Columbia, Vancouver, BC Canada; 4 Department of Pathology, British Columbia Cancer Agency, Vancouver, BC Canada; 5 Hereditary Cancer Program, British Columbia Cancer Agency, Vancouver, BC Canada

## Abstract

Germline pathogenic variants in the BRCA1-associated protein-1 (*BAP1*) gene cause the BAP1 tumor predisposition syndrome (TPDS). BAP1 TPDS is associated with an increased risk of uveal and cutaneous melanoma, mesothelioma, renal cell carcinoma, and several other cancer subtypes. Here, we report a germline nonsense *BAP1* variant (c.850G>T, p.Glu284Ter) in a patient with bladder cancer and a strong family history of malignancy. Concurrently, we identified a somatic frameshift *BAP1* variant, and as expected, immunostaining validated the loss of BAP1 protein in patient-derived tumor specimens. Together, these data provide strong evidence of pathogenicity in this case. With the addition of bladder cancer to the tumor types reported with germline *BAP1* mutations, our understanding of the BAP1 TPDS continues to evolve, and may affect future screening and surveillance guidelines.

## Introduction

Bladder cancer is the most common malignancy of the urinary tract, with an estimated 549,000 new cases diagnosed worldwide in 2018.^1^ It is characterized by one of the highest somatic mutation frequencies of any studied cancer, third only to melanoma and lung.^2^ Until recently, systemic treatment for advanced disease had been limited to cisplatin-based chemotherapy. However, a greater understanding of the molecular alterations and subtypes that define bladder cancer has resulted in a new wave of targeted therapies.^3^

In bladder cancer clinical research most next-generation sequencing (NGS) tests are aimed at identifying potentially targetable somatic alterations. However, incidental pathogenic germline variants may also be identified, even if tumor-only testing is used.^4^ The risk of incidental findings must be communicated to patients prior to consent for genomic analysis, as they confer additional risks to family members and require germline confirmation. For example, germline pathogenic variants in the *BRCA1-associated protein 1* (*BAP1*) tumor suppressor gene are the genetic cause of the BAP1 tumor predisposition syndrome (TPDS; OMIM: 614327; also known as the BAP1 cancer syndrome^5^). BAP1 TPDS is associated with an increased risk of uveal melanoma, cutaneous melanoma, mesothelioma, renal cell carcinoma, and several other cancer subtypes.^6–9^

Herein, we report a bladder cancer case with a germline *BAP1* variant, which was detected incidentally during analysis of plasma circulating tumor DNA (ctDNA).

## Results

### Case description

A 55-year-old male presented with lower urinary tract symptoms and hematuria. He was a lifelong non-smoker and his medical history was unremarkable except for nephrolithiasis. A CT scan identified a 6.4 × 7.0 × 6.7 cm fungating mass arising from the floor of the bladder and involving the ureterovesical junction bilaterally, resulting in hydronephrosis and likely muscle invasion, but no evidence of regional or distant metastatic disease. He underwent transurethral resection of bladder tumor (TURBT), which showed pT1 high grade urothelial carcinoma. He thus underwent a radical cystectomy with ileal conduit. Final pathology confirmed the initial TURBT pathology: high grade pT1 urothelial carcinoma, with lymphovascular invasion, no lymph node involvement, and negative resection margins. Incidental Gleason 3 + 3 = 6 prostatic adenocarcinoma was also detected.

He remained disease-free until 4 years later, when he re-presented with right-sided flank pain. Investigations demonstrated a new 4.6 × 4.3 cm left adrenal gland mass, a 4.7 cm mass in the right middle lobe of the lung, two lesions in the liver, a 5.7 × 4.0 × 3.5 cm soft tissue mass at L1 with impingement of the spinal cord, and widespread bony metastases. A bone biopsy of the left ulna confirmed metastatic urothelial carcinoma.

The patient was referred to our oncology centre, where he completed six cycles of cisplatin and gemcitabine chemotherapy, as well as palliative radiotherapy to the left adrenal mass, T9-L2, and left ulna. Unfortunately, 4 months after completing first-line chemotherapy, the patient had progression of bony metastases on imaging. His course was complicated by development of rapidly progressive quadriparesis secondary to a C6 metastasis, which required emergency intralesional metastatic tumor resection and cervical decompression and fixation. He passed away approximately 1 month later, at the age of 60.

### Genetic analysis

Prior to chemotherapy initiation, the patient was enrolled in a local research study developing minimally invasive prognostic and predictive genomic biomarkers. Analysis of leukocyte and plasma cell-free DNA (cfDNA) suggested a ctDNA fraction of 34.7% and revealed a hotspot somatic variant in *FGFR3* (c.746C>G, p.Ser249Cys), which is present in ~14% of all bladder cases.^10^ Additional somatic alterations included truncating mutations in *BAP1*, *KMT2D*, *EP300*, *KDM6A*, and *STAG2* (Table 1), as well as *CCND1* amplification. Interestingly, a germline nonsense *BAP1* variant, c.850G>T (p.Glu284Ter), was incidentally detected in both leukocyte DNA and cfDNA, with coverage of approximately 300× and 1600×, respectively, and is not present in the gnomAD database.^11^

**Table 1:** Germline and somatic variants identified in the proband via circulating tumor DNA analysis.

Chromosome	Gene	Variant (DNA level)	Variant (protein level)	Effect	Type
3	*BAP1*	c.1788delC	p.Ser596ArgfsTer21	Frameshift	Somatic
3	*BAP1*	c.850G>T	p.Glu284Ter	Nonsense	Germline
4	*FGFR3*	c.746C>G	p.Ser249Cys	Missense	Somatic
12	*KMT2D*	c.6973delT	p.Asp2325MetfsTer7	Frameshift	Somatic
22	*EP300*	c.63insC	p.Ala22ArgfsTer17	Frameshift	Somatic
X	*KDM6A*	c.2101_2102delTC	p.Ser701fsTer6	Frameshift	Somatic
X	*STAG2*	c.646C>T	p.Arg216Ter	Nonsense	Somatic

### Functional analysis

**Fig. 1:**
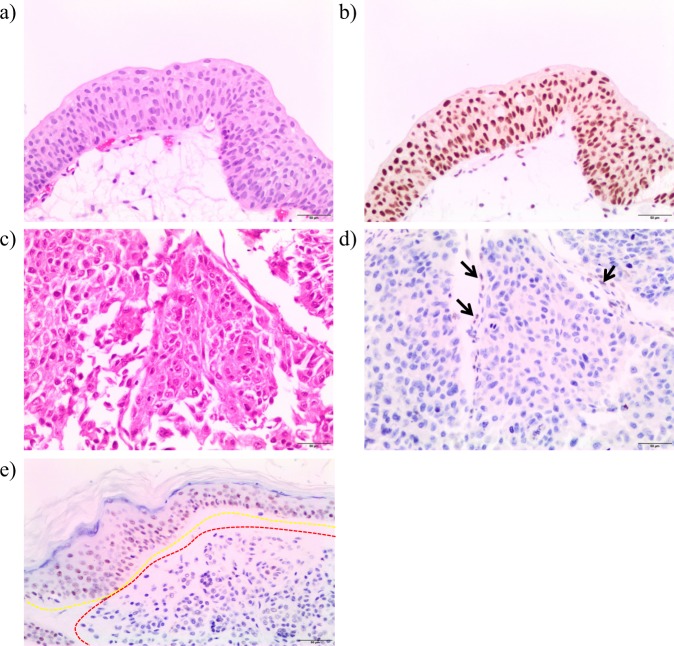
Biallelic *BAP1* mutations result in loss of protein. **a** Hematoxylin and eosin (H&E) and **b** BAP1 immunohistochemistry (IHC) showing normal urothelial histology and strong BAP1 nuclear localization, respectively. **c** H&E and **d** BAP1 IHC in the proband’s tumor showing loss of protein and weak staining of focal benign stroma cells (black arrows). **e** External control skin specimen IHC staining from a known BAP1-deficient melanoma (external negative control; red dashed line) and strong immunostaining in neighboring non-malignant tissue (external positive control; yellow dashed line). All representative images were captured at ×200 magnification. Scale bar: 50 μm.

In order to validate the loss of BAP1 protein, we performed hematoxylin and eosin (H&E) and immunohistochemistry (IHC) staining on normal (Fig. 1a, b) and malignant (Fig. 1c, d) urothelial tissue derived from the patient’s cystectomy. We observed a marked decrease in BAP1 immunostaining in the bladder tumor (Fig. 1d), compared to normal urothelium (Fig. 1b), where this protein is well-established to be highly expressed.^12,13^ In addition, IHC findings were consistent with a BAP1 external control, which was derived from a known BAP1-deficient melanoma case (Fig. 1e). Taken together, this confirms the loss of BAP1 at the protein level. As such, *BAP1* c.850G>T may be classified as a pathogenic variant, as per the American College of Medical Genetics (ACMG) guidelines (PVS1, PS3, PM2).^14^

### Family history

**Fig. 2:**
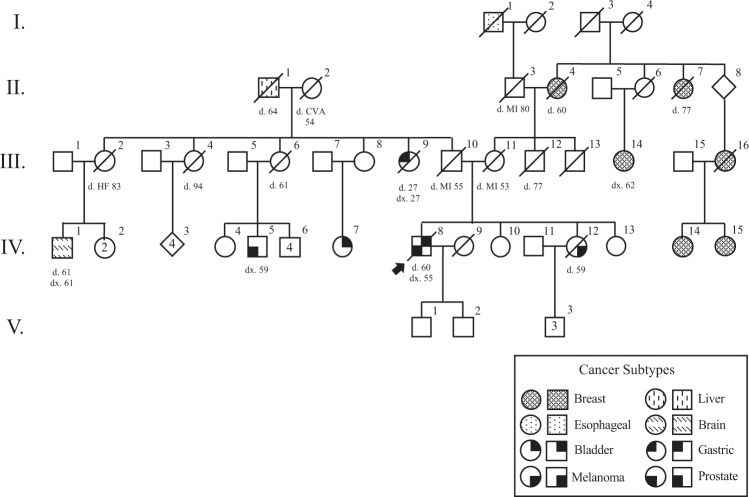
Pedigree summarizing the cancer history of the family, with proband indicated by arrowhead. Different symbols indicate tumor type, as shown in the key. HF heart failure, MI myocardial infarction, CVA stroke, dx. diagnosed, d. died.

The patient was referred to our hereditary cancer program for counseling regarding the pathogenic germline *BAP1* variant. His medical history was negative for BAP1-inactivated melanocytic nevus/melanocytoma or other cutaneous lesions, but a skin examination was not performed. Family history was notable for the proband’s sister diagnosed with melanoma of the scalp at age 52 (PID IV-12, Fig. 2). There was an extensive history of breast cancer on the proband’s maternal side, with five family members affected. The proband’s paternal aunt died of “stomach cancer” at age 27 (PID III-9) and the paternal grandfather died of “liver cancer” at age 64 (PID II-1). Three paternal cousins had a brain tumor (PID IV-1), prostate cancer (PID IV-5), and bladder cancer (PID IV-7), respectively. The proband’s family members did not consent to genetic testing.

## Discussion

We identified a germline pathogenic variant in *BAP1* in a patient with lethal bladder cancer. The variant was predicted to create a premature stop codon in exon 10 of the gene, leading to nonsense-mediated mRNA decay (NMD) or protein truncation. In addition, ctDNA analysis revealed a somatic *BAP1* frameshift mutation, also likely to result in NMD or protein truncation. *BAP1* is somatically mutated in ~3% (14/412) of localized muscle-invasive bladder cancers.^2^ Matched exome and transcriptome data from almost 10,000 human tumors revealed that *BAP1* was among the category of tumor suppressor genes exhibiting frequent NMD-triggering of heterozygous deletions, leading to biallelic inactivation by a classical “two-hit” mechanism, as seen in neurofibromatosis type-1 and malignant peripheral nerve sheath tumors.^15^ Indeed, IHC validated the functional biallelic loss of BAP1 protein, indicating that these alleles are in *trans* in the metastatic bladder cancer of this individual.

The BAP1 protein is a deubiquitinating enzyme encoded by the tumor suppressor gene located on chromosome 3p21.1. It is a binding partner to transcription factors and epigenetic chromatin remodelers, such as BRCA1 and the polycomb repressive complex, respectively. BAP1 has been implicated in several cellular processes, including cell cycle regulation, differentiation, apoptosis, DNA repair, and epigenetic histone modification.^5^ A recently described mechanism by which BAP1 exerts its tumor suppressor activity involves its dual function in the nucleus, where it regulates DNA repair, and in the cytoplasm, where it regulates apoptosis by stabilizing the inositol 1,4,5-trisphosphate receptor type 3 (IP3R3), which allows Ca^2+^ release from the endoplasmic reticulum.^16^ Thus, BAP1-deficient cells accumulate DNA damage, but are unable to execute Ca^2+^-mediated apoptosis. In addition, cells carrying heterozygous germline *BAP1* mutations have impaired mitochondrial respiration secondary to reduced Ca^2+^ levels and switch to aerobic glycolysis, which is known to promote malignant transformation and growth.^17^

Germline alterations in *BAP1* predispose to the BAP1 TPDS, an autosomal dominant syndrome encompassing uveal melanoma, cutaneous melanoma, mesothelioma and, most recently, renal cell carcinoma.^6–9,18,19^ Other cancers, such as breast and lung carcinomas, have also been reported in germline *BAP1* mutation carriers but there is insufficient data to support their inclusion in the BAP1 TPDS.^5,19^ There were no germline *BAP1* pathogenic variants identified in an analysis of 412 bladder cancer cases in The Cancer Genome Atlas.^20^ However, a germline *BAP1* splice-site variant (c.67-1G>T) has been previously reported in a patient with metastatic uveal melanoma and prior diagnosis of bladder cancer.^21^ Of note, RNA and/or protein analysis were not performed to confirm a null effect, and thus these findings must be interpreted with caution.^14^ In addition, this study only sequenced the *BAP1* gene in germline DNA, and somatic alterations were not investigated using NGS or tissue-specific IHC. Unfortunately, *BAP1* screening could not be performed in the proband’s deceased sister with cutaneous melanoma, as this malignancy is associated with the BAP1 TPDS. While the strong family history of cancers on the proband’s paternal side is suggestive of an autosomal dominance inheritance pattern, *BAP1* c.850G>T cosegregation analysis across this family would be required to validate this assumption. Identification of germline pathogenic *BAP1* variants in affected patients and families is of great clinical importance for screening and surveillance recommendations, given the diverse array of associated cancer subtypes and tendency toward tumors that are more aggressive in nature, occur at younger ages compared to the general population, and have a greater likelihood of metastasis.^5,8,9,18,19^

Interest is growing in developing therapeutic agents to reverse the phenotypic effects of BAP1 loss. For example, BAP1 regulates transcription through the post-translation modification of histones, including acetylation, suggesting a role for histone deacetylase (HDAC) inhibitors.^5^ In mesothelioma cells, BAP1 loss was found to increase HDAC1 and decrease HDAC2 expression, and sensitize to HDAC inhibitors.^22^ Although the phase III VANTAGE-014 trial comprising 661 pre-treated patients with mesothelioma did not demonstrate improved overall survival with the HDAC inhibitor vorinostat, analysis for BAP1 inactivation was not performed to determine if this subset of patients were more likely to benefit.^23^ In addition, because *BAP1* mutations lead to a deficient homologous recombination repair pathway and increase the reliance on PARP1-mediated DNA repair pathways, PARP inhibitors could induce synthetic lethality in BAP1-mutant cancers similar to BRCA1/2-mutant breast and ovarian cancers.^24^ For example, BAP1 loss sensitized to the PARP inhibitor, olaparib in cells derived from renal cell carcinoma.^24,25^ A phase II trial investigating the use of the PARP inhibitor, niraparib, in patients with tumors harboring *BAP1* and other select DNA damage response mutations is currently recruiting (NCT03207347). Likewise, various other BAP1-binding partners and downstream substrates have shown promise as drug targets, such as inhibitors of enhancer of zeste 2 polycomb repressive complex 2 subunit (EZH2)^26^ and DNA methyltransferase 1 (DNMT1).^24^

In conclusion, we present a germline nonsense *BAP1* variant in a patient with bladder cancer and a strong family history of malignancy. The patient’s concurrent somatic *BAP1* variant and the loss of BAP1 protein provides strong evidence of pathogenicity. Our understanding of the full range of malignancies associated with the BAP1 TPDS continues to evolve and may affect future screening and surveillance guidelines.

## Methods

Written informed consent was obtained for ctDNA analysis of the proband under a research protocol approved by the University of British Columbia—BC Cancer Research Ethics Board (#H16-00934). A blood sample was drawn prior to chemotherapy initiation in a Streck Cell-Free DNA BCT^®^ tube. Blood processing, and subsequent extraction of plasma cfDNA and leukocyte (germline DNA) were performed as previously described.^27,28^ We applied an established targeted DNA sequencing strategy (modified by the inclusion of a 4-bp molecular barcode to the cfDNA library’s index sequence), utilizing a custom 60-gene panel specific to urothelial carcinoma, and analyzed sequencing data as per published protocols.^27,28^ For IHC, the BAP1 primary antibody clone C4 (SC-28383, Santa Cruz Biotechnology, Mississauga, Ontario, Canada, 1:50 dilution) was used. BAP1 immunostaining was performed on deparaffinized 4-μm sections of bladder tumor on a Dako Omnis instrument (Agilent Technologies, Santa Clara, CA, USA). Control specimens consisted of sections of non-malignant proband-derived urothelium (internal control) and skin tissue from a patient with a BAP1-deficient melanoma (external control).

### Reporting summary

Further information on research design is available in the Nature Research Reporting Summary linked to this article.

## Supplementary information

Reporting Summary

## Data Availability

De-identified sequencing data that support the findings of this report have been deposited in the European Genome–phenome Archive (EGA) with the accession code “EGAS00001004055” and is available under standard EGA-controlled release.

## Code availability

Computer code for analyses performed in this study has been previously made available in Github repository https://github.com/annalam/foxa1-utr-manuscript-code.^29^ Code for structural rearrangement analysis is available in Github repository https://github.com/annalam/breakfast. Code for FASTQ/BAM file manipulation is available in Github repository https://github.com/annalam/seqkit.

## References

[1] Bray F (2018). Global cancer statistics 2018: GLOBOCAN estimates of incidence and mortality worldwide for 36 cancers in 185 countries. CA Cancer J. Clin..

[2] Robertson AG (2017). Comprehensive molecular characterization of muscle-invasive bladder. Cancer Cell.

[3] Alifrangis C, McGovern U, Freeman A, Powles T, Linch M (2019). Molecular and histopathology directed therapy for advanced bladder cancer. Nat. Rev. Urol..

[4] Raymond Victoria M., Gray Stacy W., Roychowdhury Sameek, Joffe Steve, Chinnaiyan Arul M., Parsons D. Williams, Plon Sharon E. (2015). Germline Findings in Tumor-Only Sequencing: Points to Consider for Clinicians and Laboratories: Table 1. Journal of the National Cancer Institute.

[5] Carbone M (2013). BAP1 and cancer. Nat. Rev. Cancer.

[6] Wiesner T (2011). Germline mutations in BAP1 predispose to melanocytic tumors. Nat. Genet..

[7] Testa JR (2011). Germline BAP1 mutations predispose to malignant mesothelioma. Nat. Genet..

[8] Abdel-Rahman MH (2011). Germline BAP1 mutation predisposes to uveal melanoma, lung adenocarcinoma, meningioma, and other cancers. J. Med. Genet..

[9] Popova T (2013). Germline BAP1 mutations predispose to renal cell carcinomas. Am. J. Hum. Genet..

[10] Consortium APG (2017). AACR Project GENIE: powering precision medicine through an international consortium. Cancer Discov..

[11] Karczewski, K. J. et al. Variation across 141,456 human exomes and genomes reveals the spectrum of loss-of-function intolerance across human protein-coding genes. Preprint at 10.1101/531210 (2019).

[12] Uhlen M (2015). Proteomics. Tissue-based map of the human proteome. Science.

[13] Human Protein Atlas. *Tissue Expression of BAP1: Staining in Urinary Bladder*. (2019). https://www.proteinatlas.org/ENSG00000163930-BAP1/tissue/urinary+bladder.

[14] Richards S (2015). Standards and guidelines for the interpretation of sequence variants: a joint consensus recommendation of the American College of Medical Genetics and Genomics and the Association for Molecular Pathology. Genet. Med..

[15] Lindeboom RG, Supek F, Lehner B (2016). The rules and impact of nonsense-mediated mRNA decay in human cancers. Nat. Genet..

[16] Bononi A (2017). BAP1 regulates IP3R3-mediated Ca(2+) flux to mitochondria suppressing cell transformation. Nature.

[17] Bononi A (2017). Germline BAP1 mutations induce a Warburg effect. Cell Death Differ..

[18] Carbone M (2012). BAP1 cancer syndrome: malignant mesothelioma, uveal and cutaneous melanoma, and MBAITs. J. Transl. Med..

[19] Walpole S (2018). Comprehensive study of the clinical phenotype of germline BAP1 variant-carrying families worldwide. J. Natl. Cancer Inst..

[20] Huang KL (2018). Pathogenic germline variants in 10,389 adult cancers. Cell.

[21] Turunen JA (2016). BAP1 germline mutations in Finnish patients with uveal melanoma. Ophthalmology.

[22] Sacco JJ (2015). Loss of the deubiquitylase BAP1 alters class I histone deacetylase expression and sensitivity of mesothelioma cells to HDAC inhibitors. Oncotarget.

[23] Krug LM (2015). Vorinostat in patients with advanced malignant pleural mesothelioma who have progressed on previous chemotherapy (VANTAGE-014): a phase 3, double-blind, randomised, placebo-controlled trial. Lancet Oncol..

[24] Yu H (2014). Tumor suppressor and deubiquitinase BAP1 promotes DNA double-strand break repair. Proc. Natl Acad. Sci. USA.

[25] Pena-Llopis S (2012). BAP1 loss defines a new class of renal cell carcinoma. Nat. Genet..

[26] LaFave LM (2015). Loss of BAP1 function leads to EZH2-dependent transformation. Nat. Med..

[27] Sundahl N (2019). Randomized Phase 1 trial of pembrolizumab with sequential versus concomitant stereotactic body radiotherapy in metastatic urothelial carcinoma. Eur. Urol..

[28] Vandekerkhove G (2017). Circulating tumor DNA reveals clinically actionable somatic genome of metastatic bladder cancer. Clin. Cancer Res..

[29] Annala M (2018). Frequent mutation of the FOXA1 untranslated region in prostate cancer. Commun. Biol..

